# cDC1-derived IL-27 regulates small intestinal CD4^+^ T cell homeostasis in mice

**DOI:** 10.1084/jem.20221090

**Published:** 2022-12-14

**Authors:** Fatemeh Ahmadi, Fredrik Junghus, Christian Ashworth, Amanda Lappalainen, Urs Mörbe, Knut Kotarsky, William W. Agace

**Affiliations:** 1 Immunology Section, Department of Experimental Medicine, Lund University, Lund, Sweden; 2 Mucosal Immunology Group, Department of Health Technology, Technical University of Denmark, Lyngby, Denmark

## Abstract

The small intestinal lamina propria contains large numbers of IFNγ-producing T helper (Th1) cells that play important roles in intestinal homeostasis and host defense, but the mechanisms underlying their development remain poorly understood. Here, we demonstrate that Th1 cells accumulate in the SI-LP after weaning and are maintained there long term. While both Th17 and Th1 cell accumulation in the SI-LP was microbiota dependent, Th1 cell accumulation uniquely required IL-27 and MHCII expression by cDC1. This reflected a requirement for IL-27 signaling in the priming of Th1 cells rather than for their maintenance once in the mucosa. cDC1-derived IL-27 was essential for maintaining the Th1–Th17 balance within the SI-LP, and in its absence, remaining Th1 cells expressed enhanced levels of Th17 signature genes. In conclusion, we identify cDC1-derived IL-27 as a key regulator of SI-LP Th1–Th17 cell homeostasis.

## Teaser

The underlying mechanisms that drive Th1 responses in the intestine remain unclear. Here, Ahmadi and coworkers show that the microbiota-dependent post-weaning accumulation of Th1 cells in the intestine is dependent on cDC1 expression of MHC-II and IL-27.

## Graphical Abstract

**Figure:**
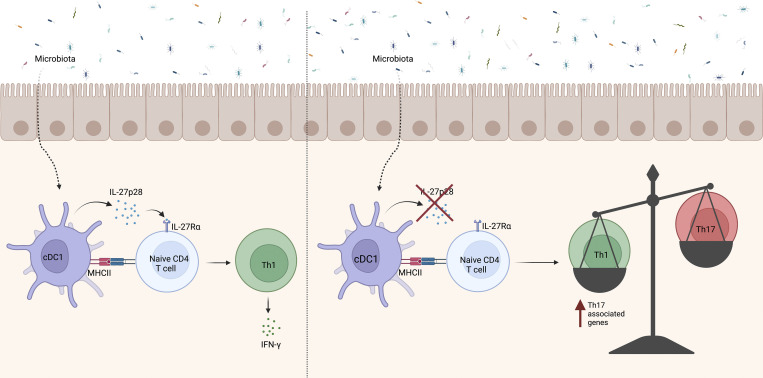

## Introduction

The small intestinal lamina propria (SI-LP) contains a large and diverse population of previously activated CD4^+^ T cells, which include prominent populations of IFN-γ–producing T helper 1 (Th1) cells and IL-17–producing Th17 cells, together with FoxP3^+^ natural (n) and induced (i) regulatory T cells (Tregs). Collectively, these cells play an essential role in maintaining tissue homeostasis and in host defense, and alterations in the composition of this compartment are associated with immune-mediated pathology and chronic intestinal inflammation (Tindemans et al., 2020). The cellular and molecular mechanisms driving the establishment and maintenance of the intestinal CD4^+^ T cell compartment remain to be fully elucidated and are likely to be different for the various CD4^+^ T cell subsets. Understanding these processes offers potential opportunities for manipulating this compartment for the benefit of human health.

Conventional dendritic cells (cDCs) are essential for CD4^+^ T cell priming and differentiation and are found throughout the intestinal LP, as well as in the intestinal inductive sites that comprise the mesenteric lymph nodes (MLNs) and the gut-associated lymphoid tissues (Joeris et al., 2017; Luciani et al., 2022). Within the LP, cDCs scan their environment for self- and foreign antigens, which they take up and present to T cells after migration to the intestinal draining MLN. cDCs consist of two major subsets, *Irf8-* and *Batf3*-dependent cDC1 that are identified by their expression of XCR1, and cDC2 that develop independently of *Irf8* and *Batf3* and express SIRPα, but not XCR1 (Anderson et al., 2021). We and others have shown that cDC1 and cDC2 play distinct non-redundant roles in intestinal T cell responses (Luda et al., 2016; Welty et al., 2013; Persson et al., 2013; Mayer et al., 2017; Demiri et al., 2017; Schlitzer et al., 2013; Lewis et al., 2011; Ohta et al., 2016; Joeris et al., 2017). For example, intestinal cDC2 appear essential for the initiation of intestinal Th2 responses (Demiri et al., 2017; Mayer et al., 2017) and intestinal LP Th17 homeostasis (Persson et al., 2013; Schlitzer et al., 2013; Welty et al., 2013; Lewis et al., 2011). In contrast, intestinal cDC1 cross-present epithelial-derived antigen to CD8^+^ T cells (Joeris et al., 2021; Cerovic et al., 2015), are important for cross-tolerance (Joeris et al., 2021), and promote the establishment of the intestinal intraepithelial lymphocyte (IEL) compartment (Luda et al., 2016; Ohta et al., 2016). In addition, our studies in *Cd11c-cre.Irf8*^*fl/fl*^ mice, which lack cDC1, have implicated a role for cDC1 in intestinal Th1 homeostasis (Luda et al., 2016). However, as *Irf8* is deleted in all CD11c-expressing cells in these mice, including intestinal macrophages and plasmacytoid DCs, the exact role of cDC1 in intestinal Th1 homeostasis and the mechanisms involved remain to be determined.

The SI-LP CD4^+^ T cell compartment develops postnatally during weaning when pups transition from a milk to solid food–based diet, and there are increases in microbial number and diversity. Establishment of the SI-LP Th17 cell compartment requires the commensal microbiota and is driven primarily by bacterial strains capable of interacting directly with the epithelium, including segmented filamentous bacteria (Ivanov et al., 2022; Goto et al., 2014; Yang et al., 2014; Ladinsky et al., 2019; Ivanov et al., 2009; Atarashi et al., 2015). In contrast, nTreg arise independently of the microbiota, and establishment of the SI-LP iTreg compartment appears to be driven primarily in response to dietary proteins (Kim et al., 2016; Tanoue et al., 2016). Whether establishment of the SI-LP Th1 compartment occurs within a similar time frame is dependent on commensal microbes, and whether Th1 cells arising during this period are maintained long term in the SI-LP remains unclear.

Here, we use *Xcr1-cre*.DTA mice to demonstrate a key role for cDC1 in the generation of microbiota-dependent SI-LP Th1 responses and identify cDC1-derived IL-27 and MHCII as key mediators in this process. Absence of cDC1-derived IL-27 led to reduced Th1 and increased Th17 cell numbers in the SI-LP and alterations in the transcriptional profile of the remaining Th1 cells, including increased transcription of Th17-associated genes. Thus, cDC1-derived IL-27 is a central regulator of SI-LP Th1/Th17 cell homeostasis.

## Results and discussion

### cDC1 and IL-27Rα play non-redundant roles in SI Th1 homeostasis

**Figure S1.**
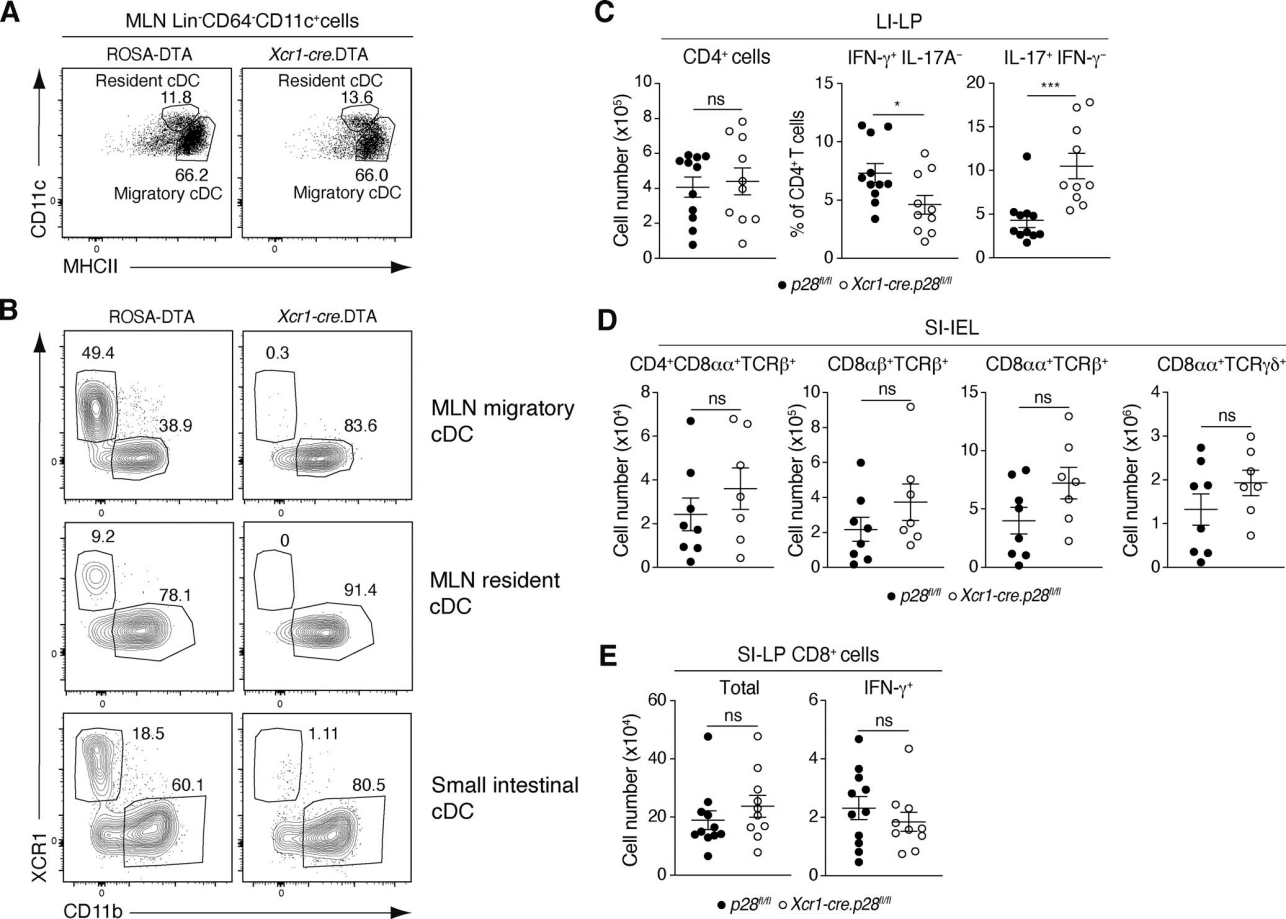
**cDC subset composition of *Xcr1-cre.DTA* mice and further characterization of the intestinal compartment of *Xcr1-cre.p28***^***fl/fl***^ **mice. (A)** Gating strategy to identify resident and migratory cDC in MLN. Cells are pregated on Lin (TCRβ, CD19, B220, and NK.1.1)^−^CD64^−^CD11c^+^ cells. **(B)** Representative flow cytometry plots of MLN and SI-LP cDC subsets in ROSA-DTA and *Xcr1-cre*.DTA mice. **(C)** Total number of CD4^+^ T cells (left panel) and proportion of IFN-γ– and IL-17A–producing CD4^+^ T cells (center and right panel) in the large intestinal (LI)-LP of adult *Xcr1-cre.p28*^*fl/fl*^ and *p28*^*fl/fl*^ control mice. **(D and E)** SI-IEL (D) and SI-LP CD8^+^ (E) subset numbers in *Xcr1-cre.p28*^*fl/fl*^ and *p28*^*fl/fl*^ control mice. **(C–E)** Results are pooled from two (D) and three (C and E) independent experiments with each symbol representing an individual mouse and lines representing mean and SEM. **(C–E)** *P < 0.05, ***P < 0.001. Mann–Whitney U test.

**Figure 1.**
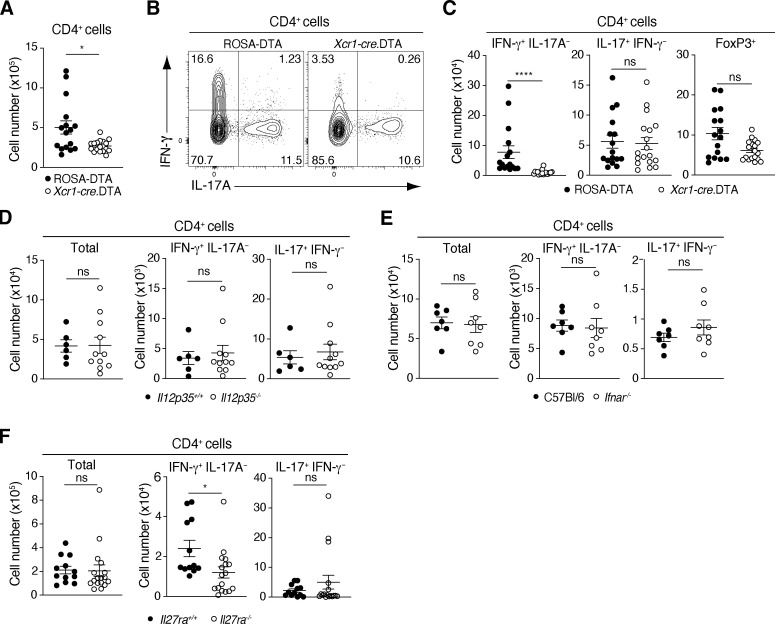
**SI-LP Th1 homeostasis requires cDC1 and IL-27Rα signaling. (A–C)** Number of CD4^+^ T cells (A), representative flow cytometry plots for IFN-γ and IL-17A staining (B), and number of IFN-γ–, IL-17A–producing, and FoxP3^+^ CD4^+^ T cells in the SI-LP (C) of ROSA.DTA and *Xcr1-cre*.DTA mice. **(D–F)** Total number of CD4^+^ T cells and IFN-γ– or IL-17–producing CD4^+^ T cells in the SI-LP of *IL12p35*^*+/+*^ and *IL12p35*^*−/−*^ mice (D), C57BL/6 and *Ifnar*^*−/−*^ mice (E), and *Il27ra*^*+/+*^ and *Il27ra*^*−/−*^ mice (F). Note, cell counts in D and E are lower as they were calculated based on bead-based counting methods after stimulation with PMA and ionomycin while those in A, C, and F were calculated based on Sysmex counts immediately after intestinal cell isolation (see Materials and methods). Results are pooled from four (A, C, and F) or two (D and E) independent experiments. Each symbol represents an individual mouse with lines representing mean and SEM. *P < 0.05, ****P < 0.0001. Mann–Whitney U test. See also Fig. S1.

To assess whether the reduction in intestinal Th1 cell numbers previously observed in *Cd11c-cre.Irf8*^*fl/fl*^ mice (Luda et al., 2016) was due to a deficiency in cDC1, *Xcr1-cre* mice (Ohta et al., 2016) were crossed with ROSA-DTA mice to selectively delete XCR1 expressing cDC1. As expected (Ohta et al., 2016), *Xcr1-cre*.DTA mice lacked cDC1 in the SI-LP and the migratory and resident compartments of draining MLN (Fig. S1, A and B). *Xcr1-cre*.DTA mice had a small but significant reduction in CD4^+^ T cell numbers in the SI-LP compared with their Cre^−^ controls (Fig. 1 A), together with a marked reduction in IFN-γ–producing Th1, but not in IL-17–producing Th17 or in Foxp3^+^ Treg numbers (Fig. 1, B and C). Thus, cDC1 play an essential role in SI-LP CD4^+^ Th1 homeostasis.

Several cytokines including IL-12, type 1 IFNs, and IL-27 have been implicated in Th1 development (Vignali and Kuchroo, 2012; Trinchieri, 2003; Yoshida and Hunter, 2015; Longhi et al., 2009), and the proportion of Th1 cells has been reported to be reduced in the colon of *Il27ra*-deficient mice (Troy et al., 2009). To address the role of these factors in SI-LP Th1 homeostasis, we assessed the SI-LP CD4^+^ T cell compartment of mice deficient in IL-12 (*Il12p35*-deficient mice), type 1 IFN signaling (*Ifnar*-deficient mice), and IL-27 receptor signaling (*Il27ra*-deficient mice). While *Il12p35-* and *Ifnar*-deficient mice had normal numbers of SI-LP CD4^+^ T cells, as well as Th1 and Th17 cells (Fig. 1, D and E), *Il27ra*-deficient mice had normal numbers of SI-LP CD4^+^ T cells and Th17 cells, but reduced numbers of Th1 cells (Fig. 1 F). Thus, IL-27, but not IL-12 or type 1 IFN, signaling plays a non-redundant role in SI-LP Th1 homeostasis.

### cDC1-derived IL-27 regulates the balance between Th1 and Th17 cells in the small intestine

**Figure 2.**
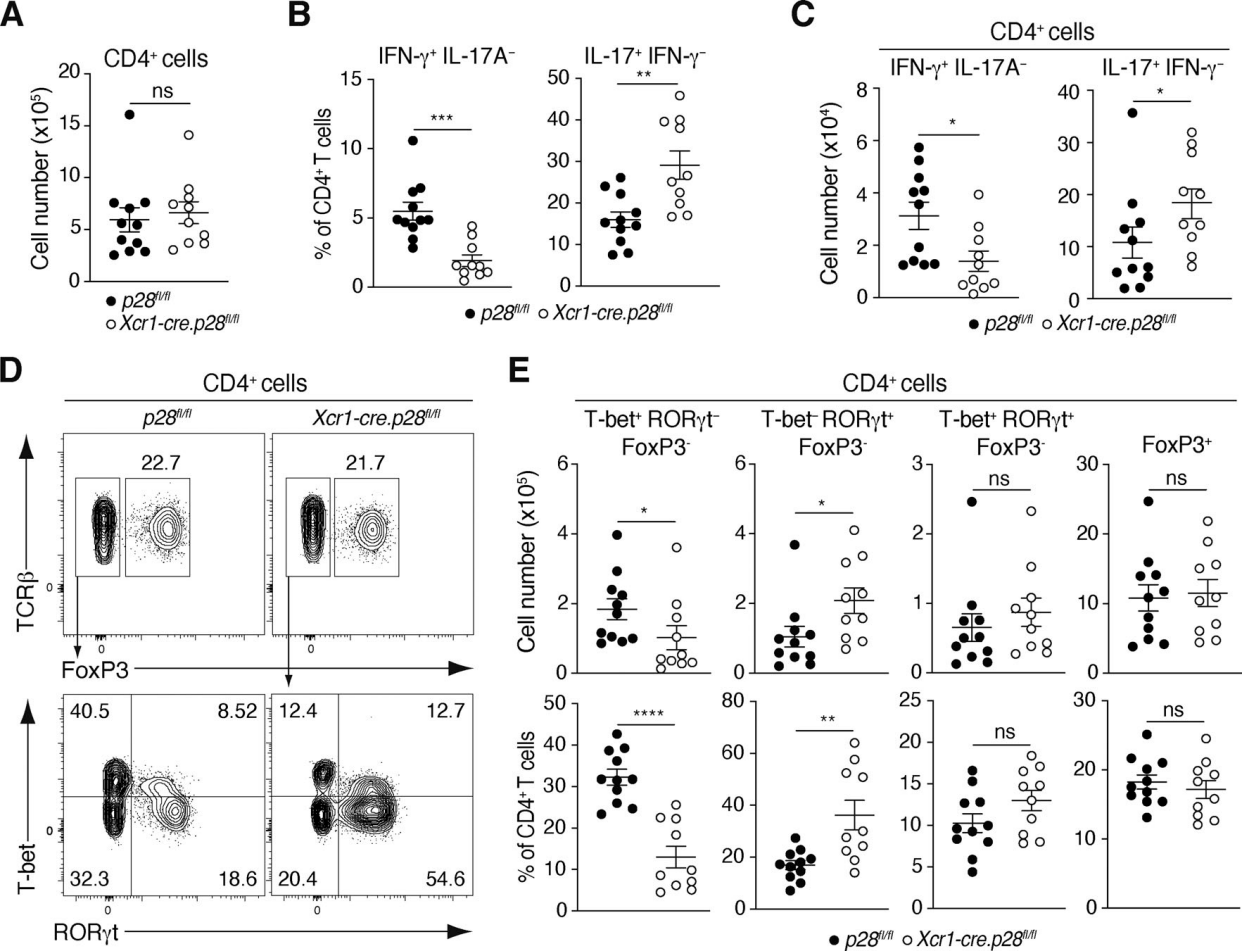
**cDC1-derived IL-27 is required for SI-LP Th1 and Th17 homeostasis. (A–C)** Number of CD4^+^ T cells (A) and percentage (B) and number (C) of IFN-γ– and IL-17A–producing CD4^+^ T cells in the SI-LP of *Xcr1-cre.p28*^*fl/fl*^ mice and *p28*^*fl/fl*^ controls. **(D and E)** Representative flow cytometry plots (D) and number (E) of CD4^+^ T cells expressing indicated transcription factors in the SI-LP. **(A–C and E)** Data are pooled from three independent experiments. Each symbol represents an individual mouse with lines representing mean and SEM. *P < 0.05, **P < 0.01, ***P < 0.001, ****P < 0.0001. Mann–Whitney U test.

While many cell types are capable of producing IL-27 (Lin et al., 2021; Kilgore et al., 2018, 2020; Hall et al., 2012b), we reasoned that the reduction of SI-LP Th1 cells in both cDC1- and *IL27ra*-deficient mice may be because cDC1-derived IL-27 is required for intestinal SI-LP Th1 homeostasis. To address this, we generated mice with a cDC1-specific deletion in the IL-27α subunit (p28/IL-30) by crossing *Xcr1-cre* mice with *p28*^*fl/fl*^ mice. *Xcr1-cre.p28*^*fl/fl*^ and *p28*^*fl/fl*^ controls had similar total numbers of SI-LP CD4^+^ T cells (Fig. 2 A), but the percentage and total number of SI-LP Th1 cells were reduced in *Xcr1-cre.p28*^*fl/fl*^ mice, while there were increased numbers of Th17 cells (Fig. 2, B and C). In line with these findings, the total number and proportion of T-bet^+^RORγt^−^FoxP3^−^ Th1 cells were reduced in the SI-LP of *Xcr1-cre.p28*^*fl/fl*^ mice compared with *p28*^*fl/fl*^ controls, while the number and proportion of RORγt^+^T-bet^−^FoxP3^−^ Th17 cells were increased (Fig. 2, D and E). In contrast, the numbers and proportions of RORγt^+^T-bet^+^FoxP3^−^ Th cells and FoxP3^+^ Treg were similar in the SI-LP of *Xcr1-cre.p28*^*fl/fl*^ and *p28*^*fl/fl*^ mice (Fig. 2 E). As with the SI-LP, *Xcr1-cre.p28*^*fl/fl*^ mice had similar total CD4^+^ T cell numbers in the large intestinal LP as *p28*^*fl/fl*^ control mice, but reduced proportions of Th1 and increased proportions of Th17 cells (Fig. S1 C). SI-LP CD8^+^ and IFN-γ^+^ CD8^+^ T cell numbers, as well as IEL numbers and subset compositions, remained unchanged in *Xcr1-cre.p28*^*fl/fl*^ mice (Fig. S1, D and E). Collectively, these results highlight a key role for cDC1-derived IL-27 in regulating the balance between Th1 and Th17 cells in the intestinal LP.

### The SI-LP Th1 compartment is established after weaning, is microbiota dependent, and is maintained long term

**Figure 3.**
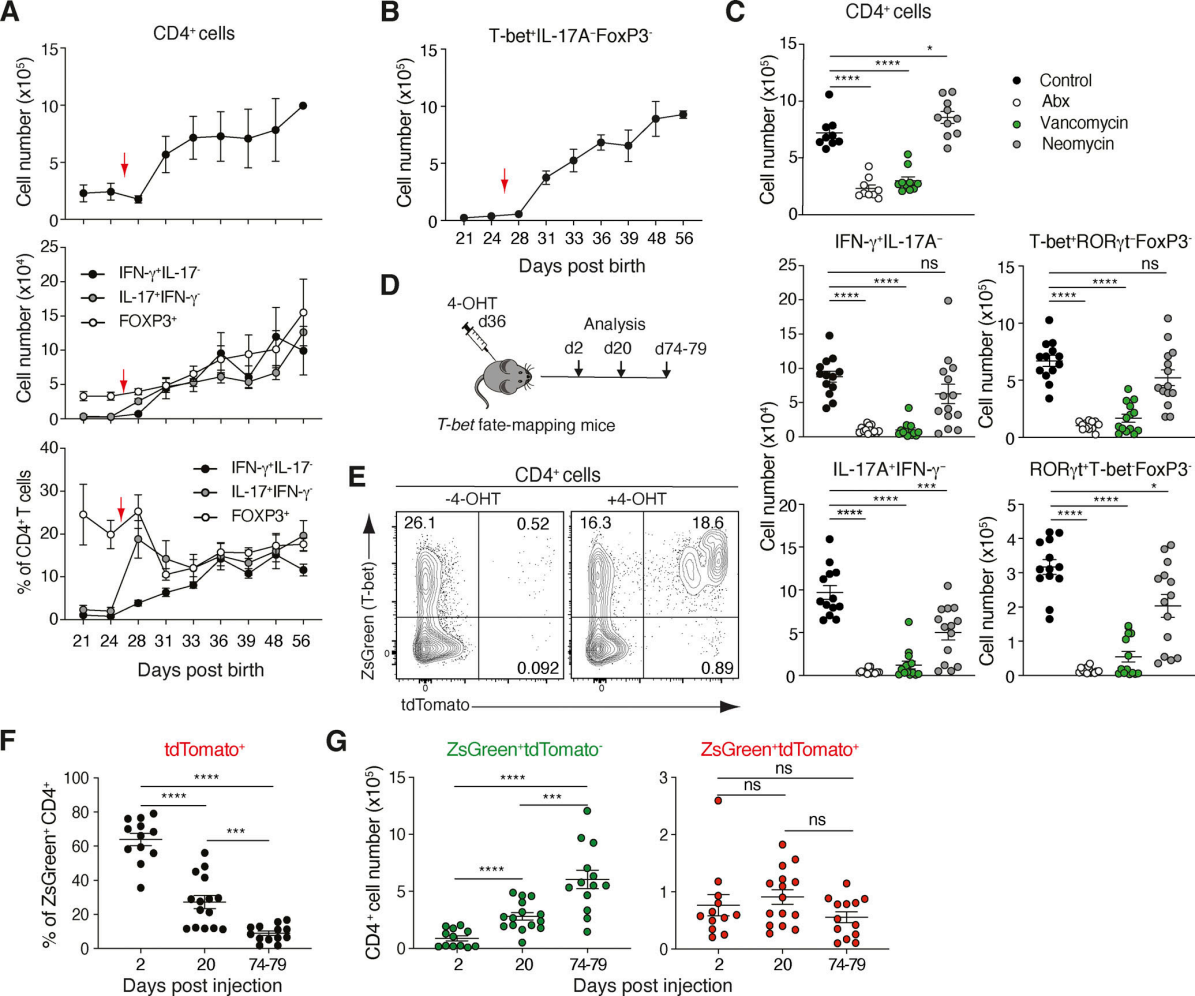
**Th1 cells accumulating in the SI-LP after weaning in response to the microbiota persist long term in the mucosa. (A and B)** Kinetics of CD4^+^ and CD4^+^ T cell subset accumulation in the SI-LP during weaning. Mice were weaned at d26 (red arrow). Results are pooled from two (A) or one (B) experiment with three to eight mice/time point. Each symbol represents mean with lines representing SEM. **(C)** Absolute number of CD4^+^, IFN-γ^+^, IL-17A^+^, T-bet^+^, or RORγt^+^ CD4^+^ T cells in the SI-LP of 33-d-old C57Bl/6 mice that received indicated antibiotics from d21 after birth. Abx, antibiotic mix (ampicillin, vancomycin, neomycin, and metronidazole). Control mice were maintained on drinking water alone. Results are from three pooled experiments with each symbol representing an individual mouse and lines representing mean and SEM. **(D)** Schematic drawing of experimental design. *T-bet* fate-mapping mice were injected with 4-OHT 36 d after birth and analyzed at indicated time points. **(E–G)** Representative flow cytometry analysis (2 d after 4-OHT injection; E), percentage of tdTomato^+^ cells amongst ZsGreen^+^ CD4^+^ T cells (F), and number of ZsGreen^+^tdTomato^-^ and ZsGreen^+^tdTomato^+^ CD4^+^ T cells in the SI-LP at indicated time points after injection of 4-OHT (G). **(F and G)** Results are from three (d2), four (d20), and three (d74–79) pooled experiments. Each symbol represents an individual mouse with lines representing mean and SEM. **(C, F, and G)** *P < 0.05, ***P < 0.001, ****P < 0.0001. Mann–Whitney U test.

Th17 cells and iTregs accumulate in the SI-LP after weaning (Atarashi et al., 2011; Ivanov et al., 2008). Whether Th1 cells accumulate in the SI-LP during this time and with similar kinetics remains unclear. To address this, we assessed the numbers of SI-LP Th1, Th17, and Treg cells in WT mice before weaning (day 21 [d21] and d24 after birth), immediately after weaning (d26), and from d28 until 8 wk of age (Fig. 3 A). Although mice start to wean from milk to solid food prior to separation from their mothers, total CD4^+^ T cell numbers increased in the SI-LP after separation, with expanded populations of Th17 cells and FoxP3^+^ Tregs (Fig. 3 A). IFN-γ– and T-bet–expressing Th1 cells also accumulated within the SI-LP during this period (Fig. 3, A and B). Thus, these CD4^+^ Th subsets accumulated in the SI-LP from weaning (d26) until d36 and their numbers appeared to stabilize somewhat thereafter. Notably, weaning was associated with a marked reduction in the proportion of FoxP3^+^ Tregs, a rapid increase in the proportion of Th17 cells, and a more gradual increase in the proportion of Th1 cells amongst the total SI-LP CD4^+^ T cell pool (Fig. 3 A), indicating that expansion of the SI-LP Th17 compartment preceded that of the Th1 compartment. To assess whether the accumulation of SI-LP Th1 cells was dependent on the microbiota, mice were given different antibiotic regimes (ampicillin, vancomycin, neomycin, and metronidazole) to deplete all bacteria, Gram-positive bacteria (vancomycin alone), or Gram-negative bacteria (neomycin alone) in their drinking water for 12 d from d21 after birth, and the SI-LP CD4^+^ T cell compartment was assessed on d33 after birth. Consistent with previous results (Ivanov et al., 2008), broad-spectrum antibiotics and vancomycin both resulted in a significant reduction in CD4^+^ T cell and Th17 cell numbers in the SI-LP, and a similar pattern was observed for IFN-γ–producing and T-bet^+^ Th1 cells (Fig. 3 C). Neomycin had little effect on total SI-LP CD4^+^ Th cell numbers, although Th17 cell numbers were significantly, albeit slightly, reduced in neomycin-treated mice. Thus, the microbiota is essential for the early life establishment of the SI-LP Th1 compartment.

To determine whether the microbiota-dependent Th1 cells that accumulate in the SI-LP after weaning are maintained long term, *T-bet* fate-mapping mice (Yu et al., 2015) were used to track the fate of T-bet–expressing CD4^+^ T cells in the SI-LP of 36-d-old mice. In these mice, ZsGreen is expressed under control of the *T-bet* promotor, rendering all T-bet–expressing CD4^+^ T cells ZsGreen^+^. Administration of 4-hydroxytamoxifen (4-OHT) to these mice drives Cre-mediated tdTomato expression in ZsGreen^+^ CD4^+^ T cells, allowing the fate of T-bet–expressing cells present at the time of injection to be traced over time (Yu et al., 2015). 2 d after 4-OHT administration to 36-d-old *T-bet* fate-mapping mice, a large proportion of ZsGreen^+^ CD4^+^ T cells in the SI-LP also expressed tdTomato, and the proportion of tdTomato^+^ZsGreen^+^ cells amongst total SI-LP ZsGreen^+^ CD4^+^ T cells then declined over the following 10 wk (Fig. 3, D–F). This proportional decline was due to a relative increase in the number of tdTomato^−^ZsGreen^+^ CD4^+^ T cells in the SI-LP and not to a loss of tdTomato^+^ZsGreen^+^ CD4^+^ T cells, whose absolute numbers remained stable (Fig. 3 G). Thus, the Th1 cells that accumulate in the SI-LP after weaning are maintained long term, although more cells of this kind continue to arrive at later times.

### Microbiota-driven establishment of the SI-LP Th1 compartment requires antigen presentation and IL-27 production by cDC1

**Figure 4.**
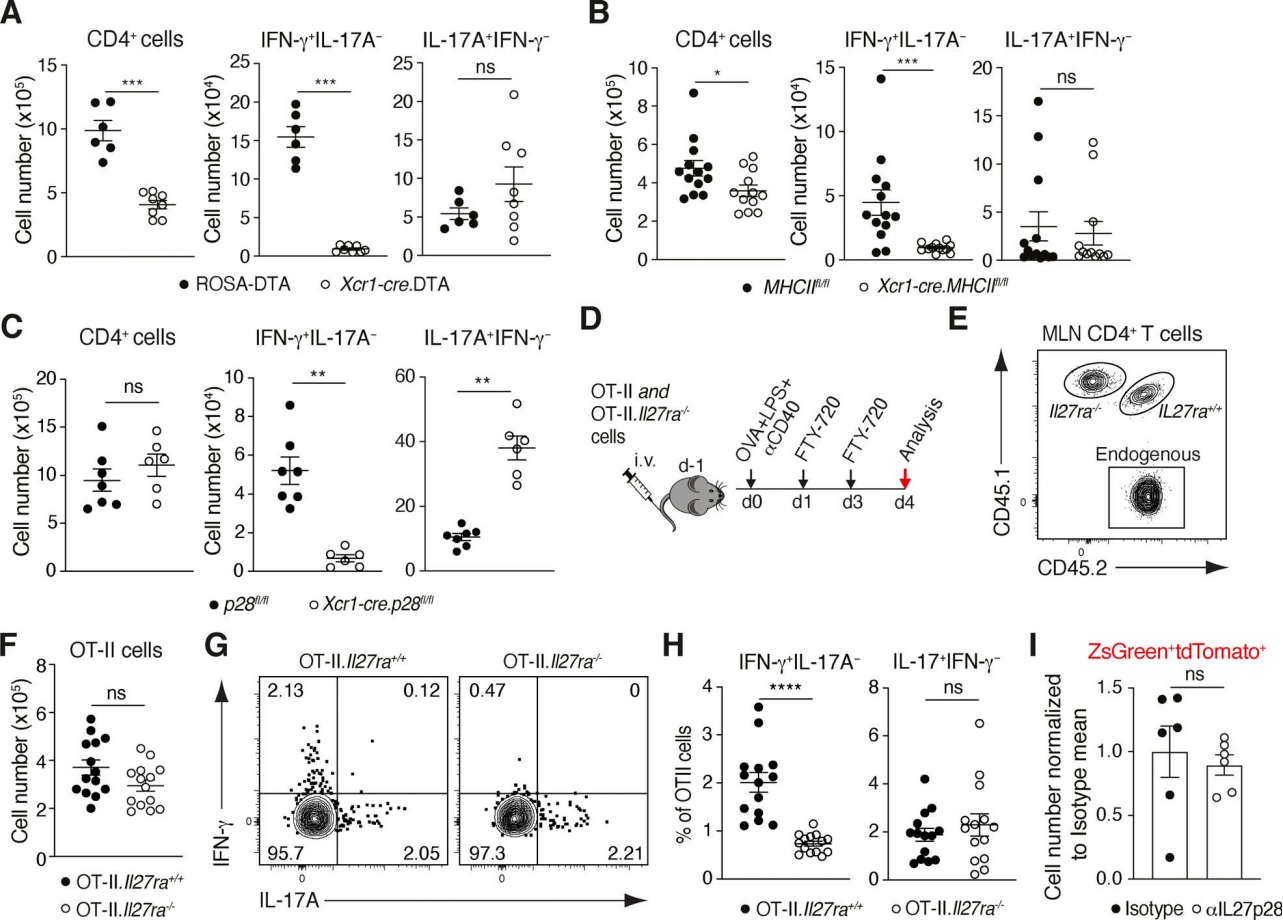
**The microbiota-dependent post-weaning accumulation of Th1 cells in the SI-LP is dependent on MHCII and p28 expression by cDC1. (A–C)** Number of SI-LP CD4^+^ T cells, IFN-γ^+^, and IL-17^+^ CD4^+^ T cells in 36-d-old ROSA.DTA and *Xcr1-cre*.DTA (A), MHCII^*fl/fl*^ and *Xcr1-cre*.MHCII^*fl/fl*^ (B), and *Xcr1-cre.p28*^*fl/fl*^ and *p28*^*fl/fl*^ mice (C). Results are pooled from two (A) and three (B and C) independent experiments with each symbol representing an individual mouse and lines representing mean and SEM. **(D–H)** *Il27ra*^*+/+*^ (CD45.1^+^CD45.2^+^) and *Il27ra*^*−/−*^ (CD45.1^+^CD45.2^−^) OT-II cells were transferred to CD45.1^−^CD45.2^+^ recipient mice at a 1:1 ratio, which were immunized i.p. with OVA, αCD40, and LPS, and MLN was analyzed 4 d after immunization. **(D)** Schematic of experimental setup. **(E)** Representative gating strategy to identify donor OT-II cells in MLN. **(F)** Number of indicated OT-II cells in MLN. **(G)** Representative flow cytometry plots. **(H)** Proportions of IFN-γ^+^ and IL-17^+^ OT-II cells. **(F and H)** Results are pooled from two independent experiments with each symbol representing an individual mouse and lines representing mean and SEM. **(I)** *T-bet* fate-mapping mice were injected with 4-OHT and indicated antibodies according to the experimental scheme are outlined in Fig. S2 J. Number of ZsGreen^+^tdTomato^+^CD4^+^ T cells in the SI-LP 14 d after the start of antibody treatment. Numbers are normalized to the mean number of ZsGreen^+^tdTomato^+^CD4^+^ T cells observed in the SI-LP of isotype control-treated mice. Results are pooled from two independent experiments with each symbol representing an individual mouse and lines representing mean and SEM. **(A–C, F, H, and I)** *P < 0.05, **P < 0.01, ***P < 0.001, ****P < 0.0001. Mann–Whitney U test. See also Fig. S2.

**Figure S2.**
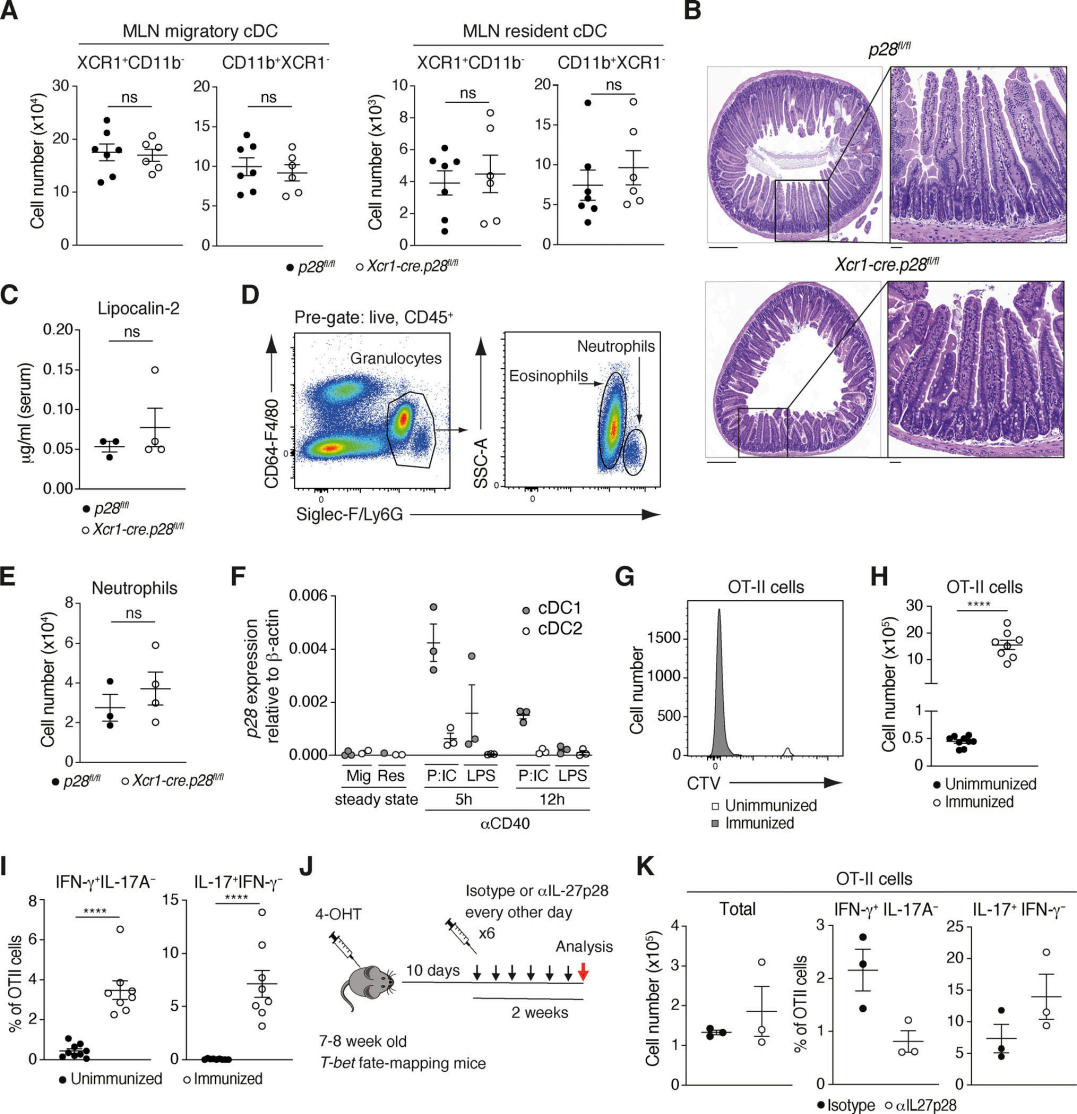
**αIL-27p28 treatment studies. (A–C)** cDC subset numbers in the MLN (A), representative H&E staining of the small intestine (B), and serum lipocalin levels (C) of 36-d-old *Xcr1-cre.p28*^*fl/fl*^ and *p28*^*fl/fl*^ control mice. **(D and E)** Representative flow cytometry staining for neutrophils (D) and total neutrophil numbers in the SI-LP (E) of 36-d-old *Xcr1-cre.p28*^*fl/fl*^ and *p28*^*fl/fl*^ control mice. **(A, C, and E)** Results are pooled from three (A) and two (C and E) experiments with each symbol representing an individual mouse and lines representing mean and SEM. **(B)** Results are representative staining of the small intestine of three *p28*^*fl/fl*^ and four *Xcr1-cre.p28*^*fl/fl*^ mice. Scale bars: left panels, 1 mm; right panels, 200 μm. **(F)** Indicated cDC subsets were sorted from the MLN of WT mice in steady state or 5 and 12 h after injection with αCD40 and LPS or poly(I:C), and expression of *p28* was assessed by real-time PCR. Results are pooled from three independent experiments with each symbol representing cDC subsets pooled from the MLN of two mice (steady state) or from an individual mouse (αCD40 and LPS or poly[I:C]-treated mice), and line represents mean and SEM. Mig, migratory; Res, resident. **(G–I)** WT recipients of Cell Trace violet (CTV)–labeled OT-II cells were immunized i.p. with OVA, αCD40, and LPS or left unimmunized according to the experimental setup in Fig. 4 D, and MLN was analyzed 4 d after immunization. Representative CTV labeling of OT-II cells (G), total OT-II cell number (H), and percentage of indicated cytokine-producing OT-II cells (I). **(H and I)** Results are from two pooled experiments with each symbol representing an individual mouse and lines representing mean and SEM. **(J)** Layout of experimental design for αIL-27p28 treatment studies in *T-bet* fate-mapping mice. *T-bet* fate-mapping mice (7–8 wk old) were injected i.p. with 4-OHT, rested for 10 d, and then injected i.p. with isotype or αIL-27p28 antibody every 2 d for 2 wk (total six injections) before analysis. **(K)** OT-II cells were transferred into recipient mice and the proportion of IFN-γ– and IL-17A–producing OT-II cells was assessed in the MLN 4 d after immunization with OVA, αCD40, and LPS. Mice received isotype or αIL-27p28 (250 μg/injection) with immunization and 2 d after immunization. Each symbol represents an individual mouse, and lines represent mean and SEM. **(A, C, E, H, I, and K)** ****P < 0.0001. Mann–Whitney U test.

To determine whether the accumulation of Th1 cells in the SI-LP after weaning was dependent on cDC1, we assessed the SI-LP CD4^+^ T cell compartment of 36-d-old *Xcr1-cre*.DTA mice. As observed in adult *Xcr1-cre*.DTA mice (Fig. 1 A), 36-d-old *Xcr1-cre*.DTA mice had a reduced total number of SI-LP CD4^+^ T cells and a marked reduction in SI-LP Th1 cells compared with Cre^−^ controls, and while Th17 numbers showed a trend toward higher numbers, this was not significant (Fig. 4 A). The optimal accumulation of SI-LP CD4^+^ T cells was also dependent on MHCII expression by cDC1 (Fig. 4 B), and more specifically, Th1 cells, as Th1, but not Th17, cell numbers were significantly reduced in 36-d-old *Xcr1-cre*.MHCII^*fl/fl*^ mice (Fig. 4 B). As observed in adult *Xcr1-cre.p28*^*fl/fl*^ mice, 36-d-old *Xcr1-cre.p28*^*fl/fl*^ mice had similar numbers of SI-LP CD4^+^ T cells, but a significant reduction in SI-LP Th1 and significant increase in SI-LP Th17 cells compared with *p28*^*fl/fl*^ control mice (Fig. 4 C). *Xcr1-cre.p28*^*fl/fl*^ mice had normal cDC subset numbers and composition in the MLN at this age (Fig. S2 A) and showed no signs of SI-LP inflammation, as assessed by histology, serum lipocalin-2 levels, and neutrophil counts in the SI-LP (Fig. S2, B–E). Thus, both MHCII and IL-27 expression by cDC1 is required for microbiota-dependent establishment of the SI-LP Th1 compartment.

To assess the role for IL-27 and IL-27 signaling in T cells during T cell priming in MLN, we first examined *p28* expression by MLN cDC. Consistent with previous reports analyzing splenic or skin draining LN cDC (Kilgore et al., 2018; Kilgore et al., 2020), MLN cDC did not express *p28* in steady state but upregulated *p28* following i.p. administration of anti-CD40 and LPS or polyinosinic–polycytidylic acid (poly[I:C]), and in particular, cDC1 (Fig. S2 F). To assess the role of IL-27 signaling in T cells during Th1 priming, OVA-specific OT-II (CD45.1^+^CD45.2^+^) and OT-II.*Il27ra*^−/−^ (CD45.1^+^CD45.2^−^) cells were coinjected into C57BL/6 (CD45.1^−^CD45.2^+^) mice at a 1:1 ratio, and the recipients were immunized i.p. with OVA, LPS, and αCD40 1 d later (Fig. 4, D and E). Recipient mice were sacrificed 4 d after immunization, and the number and cytokine profile of donor OT-II cells were assessed in the MLN by flow cytometry. As expected, transferred OT-II cells failed to expand or produce cytokines in the absence of immunization while immunization resulted in marked OT-II cell expansion and the generation of both IFN-γ– and IL-17–producing OT-II cells (Fig. S2, G–I). After immunization, similar numbers of OT-II and *Il27ra*-deficient OT-II cells were recovered from the MLN of recipient mice (Fig. 4 F), demonstrating that CD4^+^ T cell priming is not affected by the absence of IL-27 signaling in these cells. In contrast, the proportion of IFN-γ–producing, but not IL-17–producing cells, was significantly reduced in *Il27ra*-deficient OT-II cells compared with WT OT-II cells (Fig. 4, G and H). Thus, IL-27Rα expression by primed CD4^+^ T cells is required to polarize their differentiation into Th1 cells.

Given the long-term residency of SI-LP Th1 cells, we next determined whether IL27 signaling was important in maintaining these cells after their arrival in the SI-LP. To assess this, 7–8-wk-old *T-bet* fate-mapping mice were injected with 4-OHT, rested for 10 d, injected every other day for 2 wk with anti–IL-27p28 or isotype control antibody, and the number of tdTomato^+^ZsGreen^+^ cells determined in the SI-LP 2 d after the final injection (Fig. S2 J). Anti–IL-27p28 inhibited OT-II differentiation into Th1 cells in the MLN following i.p. immunization with OVA, anti-CD40, and LPS (Fig. S2 K), demonstrating its neutralizing activity in vivo. However, anti–IL-27p28 had no impact on the numbers of tdTomato^+^ZsGreen^+^ CD4^+^ T cells in the SI-LP (Fig. 4 I). Collectively, these results indicate that the principal role of IL-27 is in the initial polarization of Th1 cells and not in their maintenance after their arrival in the SI-LP.

### cDC1-derived IL27 regulates the transcriptional profile of SI-LP Th subsets

**Figure 5.**
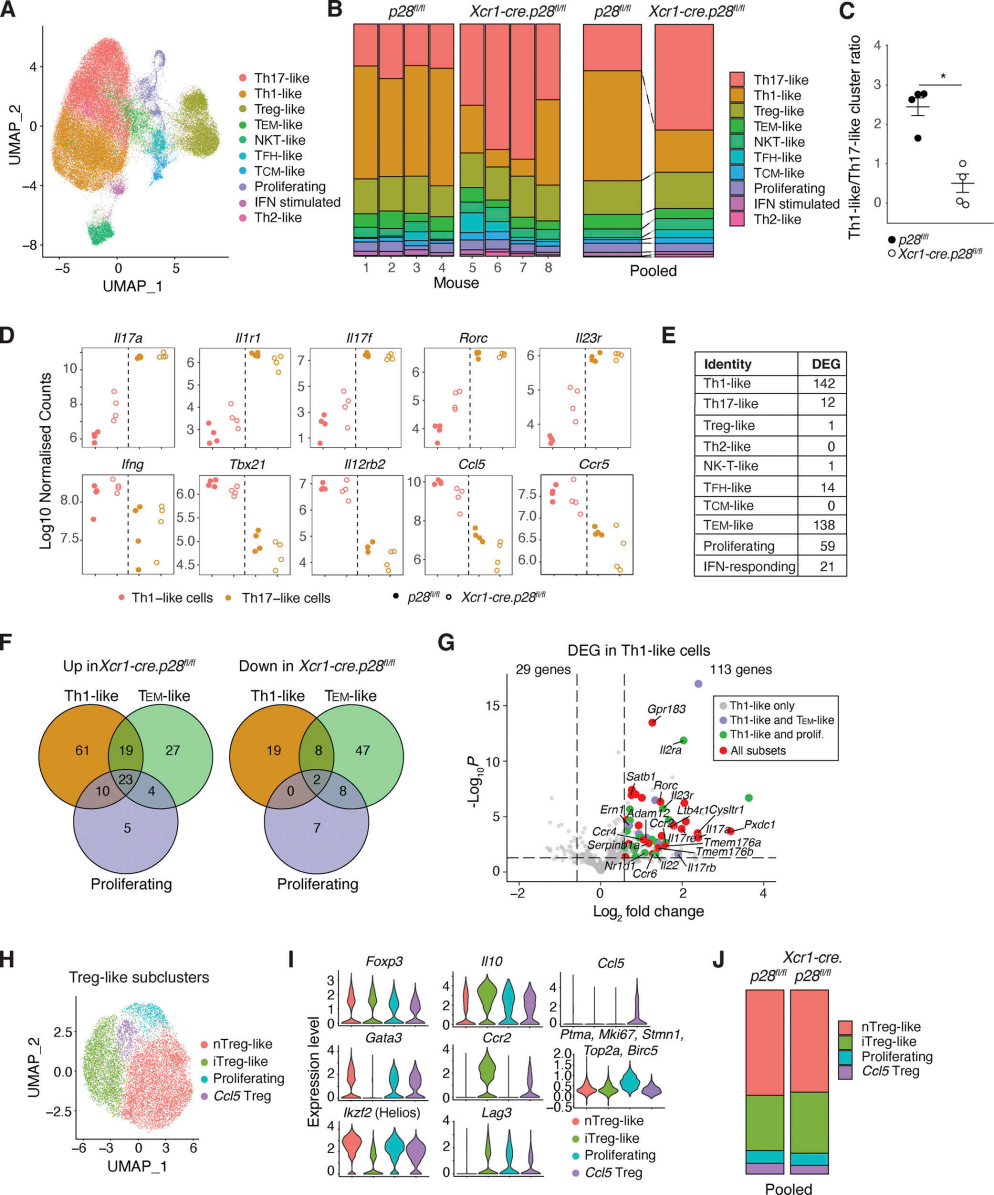
**cDC1-derived IL-27 regulates the proportions and transcriptional profile of SI-LP CD4**^**+**^ **T cell subsets. (A)** Combined UMAP of scRNA-seq data of flow cytometry sorted SI-LP CD4^+^ T cells from 8-wk-old male *Xcr1-cre.p28*^*fl/fl*^ and *p28*^*fl/fl*^ mice. The identity of clusters is based on DEGs shown in Fig. S3 B. **(B)** Proportions of each cluster in individual (left panel) or pooled (right panel) *Xcr1-cre.p28*^*fl/fl*^ and *p28*^*fl/fl*^ mice. **(C)** Ratio of Th1-like to Th17-like cells in indicated mice. Each symbol represents an individual mouse and lines represent mean and SEM. *P < 0.05, Mann–Whitney U test. **(D)** Expression of individual Th1 and Th17 signature genes in Th1- and Th17-like clusters from *Xcr1-cre.p28*^*fl/fl*^ and *p28*^*fl/fl*^ mice. Each symbol represents log10-normalized pseudobulk counts of the indicated cluster from an individual mouse. **(E)** Number of DEGs in each Th cell cluster between *Xcr1-cre.p28*^*fl/fl*^ or *p28*^*fl/fl*^ mice. Results are based on pseudobulk analysis of scRNA-seq data (P < 0.05, log_2_fold change >0.58, and selecting genes that were up or down in all *Xcr1-cre.p28*^*fl/fl*^ mice compared with *p28*^*fl/fl*^ mice). **(F)** Venn diagram showing number of overlapping genes that are up or down in the indicated SI-LP CD4^+^ T cell subset of *Xcr1-cre.p28*^*fl/fl*^ mice. **(G)** Volcano plot of DEG in SI-LP Th1-like cells of *Xcr1-cre.p28*^*fl/fl*^ and *p28*^*fl/fl*^ mice. Colored dots represent genes that are also upregulated in CD4^+^ T cell subsets in F, as indicated. Named genes are associated with Th17 cells. **(H)** UMAP of SI-LP CD4^+^ Treg clusters. **(I and J)** Violin plots of indicated gene expression by each SI-LP Treg cluster (I) and proportion of each Treg cluster amongst total SI-LP Tregs of *Xcr1-cre.p28*^*fl/fl*^ and *p28*^*fl/fl*^ mice (J). See also Fig. S3. T_EM_, effector memory T cell; NKT, natural killer T cell; T_FH_, follicular helper T cell; T_CM_, central memory T cell.

**Figure S3.**
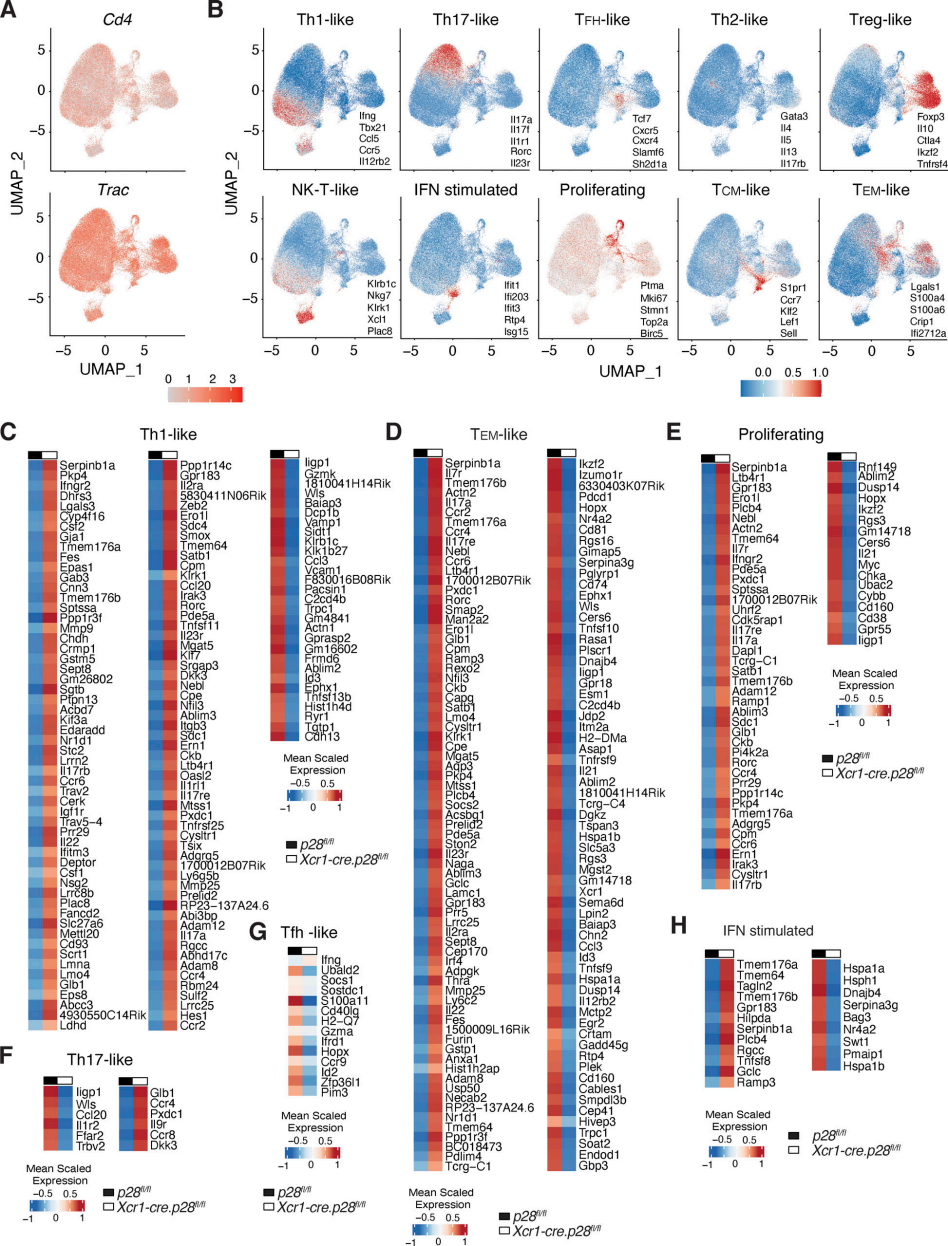
**Determination of SI-LP Th cluster identities. (A)** Combined UMAP of scRNA-seq data of flow cytometry sorted SI-LP CD4^+^ T cells from 8-wk-old *Xcr1-cre.p28*^*fl/fl*^ and *p28*^*fl/fl*^ controls, depicting expression of *Cd4* and *Trac* across clusters. **(B)** UMAP cluster of pooled CD4^+^ T cells from *Xcr1-cre.p28*^*fl/fl*^ mice and *p28*^*fl/fl*^ controls, depicting expression levels of indicated lineage genes across clusters to determine cluster identity. **(C–H)** Heat map of DEGs in indicated SI-LP CD4^+^ T cell subsets between *Xcr1-cre.p28*^*fl/fl*^ mice and *p28*^*fl/fl*^ controls. Mean scaled expression per cell was calculated for each cluster across pooled *Xcr1-cre.p28*^*fl/fl*^ mice and *p28*^*fl/fl*^ controls.

To assess the broader impact of cDC1-derived IL-27 on the properties of SI-LP CD4^+^ T cells, these cells were sorted from *Xcr1-cre.p28*^*fl/fl*^ mice and *p28*^*fl/fl*^ controls (four mice/group) and subjected to single-cell RNA sequencing (scRNA-seq). After bioinformatically removing naive CD4^+^ T cells, sequences were obtained for 39,013 SI-LP CD4^+^ T cells from *Xcr1-cre.p28*^*fl/fl*^ mice and 35,915 cells from *p28*^*fl/fl*^ mice. Louvain clustering identified 10 *Cd4*^*+*^ *Trac* (TCRα constant)^+^ clusters (Fig. 5 A and Fig. S3 A), which comprised distinct populations of CD4^+^ T cells based on their differential expression of Th subset lineage genes (Fig. S3 B). The relative proportions of these clusters differed between *Xcr1-cre.p28*^*fl/fl*^ and *p28*^*fl/fl*^ mice, with the former having decreased proportions of Th1-like and increased proportions of Th17-like cells (Fig. 5, B and C), consistent with earlier flow cytometry analysis (Fig. 2). In contrast, the proportions of other SI-LP CD4^+^ T cell clusters did not differ between these mice (Fig. 5 B). These results are consistent with previous studies indicating a role for IL-27 in the promotion of Th1 and inhibition of Th17 responses (Mei et al., 2021; Hall et al., 2012b; Yoshida and Hunter, 2015) and highlight an essential role for cDC1-derived IL-27 in these processes within the intestine. Of note, in contrast to *Xcr1-cre.p28*^*fl/fl*^ mice, we did not observe an increase in SI-LP Th17 numbers in *Xcr1-cre*.DTA and *IL27ra*-deficient mice (Fig. 1, C and F). Given the complexity of both IL-27 and cDC1 biology, further studies will be required to dissect the mechanisms underlying these apparently discrepant findings.

As expected, analysis of expression levels of individual Th1 and Th17 signature genes demonstrated that Th17-like cells expressed high levels of Th17-associated but low levels of Th1-associated signature genes, and the expression levels of these signature genes did not differ between *Xcr1-cre.p28*^*fl/fl*^ mice and *p28*^*fl/fl*^ controls (Fig. 5 D). In contrast, while Th1-like cells from *Xcr1-cre.p28*^*fl/fl*^ and *p28*^*fl/fl*^ mice expressed similar levels of Th1 signature genes, Th1-like cells in *Xcr1-cre.p28*^*fl/fl*^ mice expressed higher levels of Th17 signature genes compared with Th1-like cells from *p28*^*fl/fl*^ mice (Fig. 5 D), although not to the level observed in Th17-like cells. Differentially expressed gene (DEG) analysis demonstrated that the transcriptional profile of several SI-LP CD4^+^ T cells subsets differed between *Xcr1-cre.p28*^*fl/fl*^ mice and *p28*^*fl/fl*^ controls (Fig. 5 E and Fig. S3, C–H). As most of these DEGs were concentrated within the clusters containing Th1-like, T effector memory–like, and proliferating CD4^+^ T cell clusters, we assessed whether there were any up- or downregulated genes that were shared across these subsets (Fig. 5 F). Each subset expressed a higher number of upregulated genes in *Xcr1-cre.p28*^*fl/fl*^ mice compared with downregulated genes, with 23 of these being shared by all subsets and a further 29 shared between Th1-like cells and either T effector memory–like or proliferating CD4^+^ T cells (Fig. 5 F). In contrast, few of the downregulated genes in *Xcr1-cre.p28*^*fl/fl*^ mice were shared by the different subsets (Fig. 5 F). Notably, many of the shared upregulated genes were genes normally associated with Th17 cell differentiation and function (Fig. 5 G). Thus, in addition to regulating the Th1/Th17 balance within the intestine, cDC1-derived IL-27 plays an important role in inhibiting the expression of Th17-associated genes in developing Th1 cells.

Since numerous reports have suggested a role for IL-27 in the generation and function of Treg (Zhang et al., 2020; Hall et al., 2012b; Do et al., 2016; Hall et al., 2012a), we further investigated the role of cDC1-derived IL-27 on intestinal Treg subset composition and transcription. For this, Treg-like cells from *Xcr1-cre.p28*^*fl/fl*^ and *p28*^*fl/fl*^ SI-LP were bioinformatically reisolated and subclustered (Fig. 5, H and I). This identified four FoxP3^+^ Treg subclusters: a major cluster of *Ikzf2*^hi^*Gata3*^hi^ nTreg-like cells, a major cluster of *Il-10*^hi^*Lag3*^hi^*Ccr2*^hi^ iTreg-like cells, a small cluster of proliferating Tregs, and a minor cluster of *Ccl5*-expressing Tregs that also selectively expressed *Ifng* and *Cxcr6*, like a recently described population of *Ifng*^+^ Treg found in spleen, lung, and colon (Lu et al., 2020). The proportions of these subclusters did not differ between *Xcr1-cre.p28*^*fl/fl*^ and *p28*^*fl/fl*^ mice (Fig. 5 J), and only nTregs and *Ccl5* Tregs showed any DEGs (five and one gene, respectively) between *Xcr1-cre.p28*^*fl/fl*^ *mice* and *p28*^*fl/fl*^ mice. Thus, cDC1-derived IL-27 does not impact SI-LP Treg subset homeostasis or transcription.

In summary, our results identify cDC1-derived IL-27 as a key determinant of SI-LP Th1/Th17 homeostasis and indicate that one of the main functions of cDC1-derived IL-27 is to promote Th1 differentiation and suppress Th17-associated transcriptional programs during microbiota-dependent T cell priming in intestinal lymphoid tissues. The long-term consequences of lacking cDC1-derived IL-27 on intestinal homeostasis and responses to intestinal inflammation/infection await further study.

## Materials and methods

### Mice

*T-bet-ZsGreen-cre* (*T-bet-ZsGreen-T2A-CreER*^*T2*^; Yu et al., 2015), *Rosa26*^*lsl-Tomato/+*^ (Säwen et al., 2018), *Il12p35*^*−/−*^ (B6.129S1-*Il12a*^*tm1Jm*^/J [#002692; JAX stock]; Mattner et al., 1996), *Il27ra*^*−/−*^ (B6N.129P2-*Il27ra*^*tm1Mak*^/J [#018078; JAX stock]; Yoshida et al., 2001), C57Bl/6NRj (Janvier Labs), *Xcr1-cre* (*B6-Xcr1*^*tm2Ciphe*^; Wohn et al., 2020), ROSA-DTA (B6.129P2-*Gt(ROSA)26Sor*^*tm1(DTA)Lky*^/J; #009669; JAX stock; Voehringer et al., 2008), MHCII^fl/fl^ (B6.129X1-*H2-Ab1*^*tm1Koni*^/J; Hashimoto et al., 2002), *Ifnar*^*−/−*^ (B6(Cg)-*Ifnar1*^*tm1.2Ees*^/J), C57BL/6-*Gt(ROSA)26Sor*^*tm1(FLP1)Dym*^ (Flp-deleter), and C57BL/6-Tg(TcraTcrb)425Cbn (OT-II) were bred and maintained at the Lund University Biomedical Centre or the Clinical Research Centre, Malmö. For the generation of *Xcr1-cre.p28*^*fl/fl*^ mice, homozygous *Il27*^*tm1a(EUCOMM)Wtsi*^ sperm (European Mouse Mutant Archive) was used to generate heterozygous *Il27*^*tm1a(EUCOMM)Wtsi*^ offspring and crossed with Flp-deleter mice to remove the neomycin cassette. The resulting *p28*^*fl/fl*^ mice were then crossed with *Xcr1-cre* mice. Animal experiments were performed in accordance with the Lund/Malmö Animal Ethics Committee. Littermates were used for all experiments except those involving *Ifnar*^*−/−*^ mice, where *Ifnar*^*−/−*^ and control C57Bl/6 mice were cohoused for 10–14 d before use.

### Mouse genotyping

DNA extracted from ear biopsies was used for genotyping using the following primers: *Xcr1-cre*, fw 5′-CCT​TCT​CCT​ACG​ACA​TTC​TGA​C-3′ and rev 5′-ACT​TCA​CCT​TCA​CGA​TGC​C-3′; ROSA-DTA, fw 5′-AAA​GTC​GCT​CTG​AGT​TGT​TAT-3′, 5′-GCG​AAG​AGT​TTG​TCC​TCA​ACC-3′ and rev 5′-GGA​GCG​GGA​GAA​ATG​GAT​ATG-3′; *ZsGreen*, fw 5′-CAG​AGA​AAG​CCC​AGG​AGC​AG-3′ and rev 5′-CCT​TGA​AGG​GGT​AGC​CGA​TG-3′; *tdTomato*, fw 5′-GGC​ATT​AAA​GCA​GCG​TAT​CC-3′ and rev 5′-CTG​TTC​CTG​TAC​GGC​ATG​G-3′; *Il12p35*, WT fw 5′-ACG​GCT​ATG​CCC​GTT​TCA​C-3′ and rev 5′-GGT​ACT​GAC​CTC​CCT​CCA​CA-3′, KO fw 5′-TGC​CGA​ATA​TCA​TGG​TGG​AAA​AT-3′ and rev 5′-TCG​TCA​AGA​AGG​CGA​TAG​AAG​GCG-3′; *Il27ra*, WT fw 5′-CAA​GAC​CTT​GTG​TGC​AGG​TG-3′ and rev 5′-GTC​ACC​ATC​TTG​AGC​CCA​GT-3′, KO fw 5′-CTT​GGG​TGG​AGA​GGC​TAT​TC-3′ and rev 5′-AGG​TGA​GAT​GAC​AGG​AGA​TC-3′; *p28*, WT, floxed, and deleted alleles, fw 5′-TCT​CTG​TAA​GTG​AAC​GGC​AAG​G-3′, fw 5′-AAG​GCG​CAT​AAC​GAT​ACC​AC-3′ and rev 5′-CCA​CTC​CCA​TCA​CAG​TCG​TC-3′; and MHCII floxed and deleted alleles, fw 5′-CAT​TTC​CTT​TGG​GTT​GCA​GT-3′ and rev 5′-CTC​ACC​CCA​GGG​TAT​GTA​TCA-3′.

### Cell isolation

LP cell suspensions were generated as described previously (Luda et al., 2016). Briefly, after removal of Peyer’s patches and fat, tissue was cut longitudinally into 1-cm pieces and incubated in HBSS (Gibco) containing Hepes (15 mM; Gibco), EDTA (2 mM), and FBS (2%; Sigma-Aldrich) for 10 min at 37°C and for colonic tissues with agitation at 370 rpm. This step was repeated two times with incubation for 15 min at 37°C. Epithelial cells and debris were removed by filtering through a nylon filter (250 μm) after each wash. The remaining tissue was washed and incubated for 20–35 min in R10 media (RPMI 1640, FBS [10%], Hepes (10 mM), sodium pyruvate [1 mM], penicillin [100 U/ml], streptomycin [100 μg/ml]; Gibco) containing 2-ME (50 μM, Gibco), DNase I (30 μg/ml; Sigma-Aldrich), and Liberase (0.3 Wuensch U/ml; Sigma-Aldrich) on a magnetic stirring plate at 37°C with 5% CO_2_. The resulting cell suspensions were filtered through a cell strainer (100 μm; Thermo Fischer Scientific) prior to density gradient centrifugation with 40–70% Percoll (GE Healthcare). For generation of MLN and splenic cell suspensions, organs were mashed through a 70-μm cell strainer.

### Flow cytometry

Flow cytometry was performed according to standard procedures using the following antibodies: CD45 (1372.3 or 30-F11), TCRβ (H57-597), CD4 (RM4-5), IA/IE (MHCII, M5/114.15.2), and RORγt (Q31-378) were from BD Bioscience; CD45.1 (A20), CD45.2 (104), TCR Vα2 (B20.1), CD4 (GK1.5), CD8α (53–6.7), CD19 (6D5), CD64 (X54-5/7.1), B220 (RA3-6B2), NK1.1 (PK136), XCR1 (ZET1), IFN-γ (XMG1.2), and IL-17A (TC11-18H10.1) were from BioLegend; CD11c (N418 ), FoxP3 (FJK-16s), and T-bet (eBio4B10) were from eBioscience; and CD11b (M1/70) was from Invitrogen. Intracellular staining was performed using the FoxP3 Fixation/Permeabilization Kit (eBioscience). For transcription factor staining, cells were first stained for surface antigens, fixed for 1 h on ice, and washed with wash buffer before staining for transcription factors on ice for 1 h. For intracellular cytokine and FoxP3 staining, cells were stained for surface antigens, fixed for 20 min at room temperature (RT), and incubated for 15 min at RT in wash buffer containing 2% rat serum to block unspecific antibody binding. Cells were then incubated with cytokine and FoxP3 antibodies overnight at 4°C. Data were acquired on a Fortessa or LSRII (BD Bioscience) or Cytek Aurora and analyzed using FlowJo software (Tree Star). Cell aggregates were excluded based on their FSC-A and FSC-W, and dead cells excluded based on positive staining for propidium iodide (Sigma-Aldrich), fixable Viability Dye eFluor 450 (Thermo Fisher Scientific), Fixable Viability Dye eFlour 780 (Thermo Fisher Scientific), or by LIVE/DEAD Fixable Aqua Dead Cell Stain Kit (Thermo Fisher Scientific).

### Assessment of *p28* expression by MLN cDC subsets

cDC subsets were sorted from the MLN of WT mice in steady state or following i.p. injection of αCD40 (25 μg; Biolegend) and LPS (20 μg; Sigma-Aldrich) or Poly(I:C) (100 μg; InvivoGen) using a FACSAria III Cell Sorter (BD Bioscience). Briefly, MLN CD11c^+^ cells were enriched using anti-CD11c magnetic cell separation beads (Miltenyi Biotec) and then stained for surface markers prior to sorting. Total RNA was isolated with an RNA Nano-prep kit (Agilent), cDNA was prepared and amplified using Ovation Pico WTA systemV2 (TECAN), and quantitative PCR to quantify *p28* expression was performed with Kapa SYBR FAST (Sigma-Aldrich). The following primers were used: b-actin sense, 5′-CCG​GGA​CCT​GAC​AGA​CTA-3′; b-actin antisense, 5′-GTT​TCA​TGG​ATG​CCA​CAG​GAT-3′; p28 sense, 5′-ATC​TCG​ATT​GCC​AGG​AGT​GA-3′; and p28 antisense, 5′-GTG​GTA​GCG​AGG​AAG​CAG​AGT-3′ (Hooper et al., 2017).

### In vivo studies

For lineage tracing experiments, *T-bet* fate-mapping mice were injected with 4-OHT (1.3 mg/mouse; Sigma-Aldrich) in a PBS containing Kolliphor (25%; Sigma-Aldrich) and ethanol (25%; Thermo Fischer Scientific) in a total volume of 130 μl/mouse. For IL-27 neutralization studies, *T-bet* fate-mapping mice were injected i.p. with anti-mouse IL-27p28 (MM27-B1) or mouse IgG2a isotype control (C1.18.4) antibody (250 μg/injection; BioXCell) every 2 d for 2 wk. To deplete the microbiota, mice received broad-spectrum antibiotics (ampicillin [1 g/liter], vancomycin [0.5 g/liter], neomycin [1 g/liter], and metronidazole [1 g/liter]), vancomycin alone (0.5 g/liter), or neomycin alone (1 g/liter) in drinking water for the indicated period, with the antibiotic solution being replaced every 2–3 d, and the control group receiving fresh water alone.

### Adoptive T cell transfers

Naive CD4^+^ T cells were purified from the MLN and spleen of OT-II and OT-II.*Il27ra*^*−/−*^ mice using the EasySep Mouse Naive CD4^+^ T cell isolation Kit (Stem Cell Technologies), according to the manufacturer’s instructions. OT-II cells (1 × 10^6^ cells/mouse) were injected i.v. into C57/BL6 recipients, and recipients were immunized 24 h later by i.p. injection of OVA (0.5 mg, Grade V; Sigma-Aldrich), anti-CD40 (25 μg), and LPS (20 μg). 1 and 3 d after immunization, recipients were injected i.p. with FTY720 (fingolimod, 20 μg; Cayman) to prevent lymphocyte egress from the MLN. Mice were sacrificed 4 d after immunization and MLN isolated for analysis. For antibody treatment studies, anti-mouse IL-27p28 or mouse IgG2a isotype control antibody were injected i.p. (250 μg) at immunization and 2 d after immunization.

### Ex vivo T cell stimulation

SI-LP or MLN cells were incubated in 5-ml round bottom tubes (1–1.2 × 10^7^ cells/tube) in R10 medium (1 ml) supplemented with PMA (25 ng/ml; Sigma-Aldrich) and ionomycin (500 ng/ml; Sigma-Aldrich) for 4 h at 37°C and 5% CO_2_. Golgi Stop (1 μl; BD Bioscience) was added to the tubes for the last 3 h to inhibit protein transport. Cell numbers were calculated for each experiment using a Sysmex cell counter (Sysmex kx-21n, Sysmex) or, where indicated, SPHERO AccuCount fluorescent particles (Spherotech). For the former, cell counts were based on Sysmex counts prior to T cell stimulation, and for the latter, 20,000 AccuCount fluorescent particles were added to each sample after T cell stimulation, and cell counts were based on bead numbers after cell surface staining, fixation, and intracellular staining using flow cytometry.

### Cell preparation for scRNA-seq

CD4^+^ T cells were enriched from SI-LP cell suspensions using the EasySep Mouse CD4^+^ T cell isolation Kit (Stem Cell Technologies) according to the manufacturer’s instructions, and CD4^+^TCRαβ^+^ T cells were sorted using a FACSAria II Cell Sorter (BD Bioscience) and subjected to droplet-based scRNA-seq using the Chromium Single Cell 3′ Reagent Kit v3 following the manufacturer’s instructions (10x Genomics). The 10x Chromium Controller generated Gel Bead-In Emulsions, where each cell was labeled with a specific barcode and each transcript labeled with a unique molecular identifier (UMI). At RT, Gel Bead-In Emulsions were broken down, and barcoded cDNA was purified with Dynabeads MyOne Silane beads (Thermo Fisher Scientific) and amplified by PCR. Amplified cDNA was cleaned up with SPRIselect (Beckman Coulter). Indexed sequencing libraries were constructed by enzymatic fragmentation, end-repair, and A-tailing before a second and final PCR amplification using the Chromium i7 Sample Index (10x Genomics), introducing an Illumina R2 sequence, a unique sample index (allowing multiplex sequencing), and P5/P7 Illumina sequencing adaptors to each library. Multiplexed libraries were pooled, and sequencing was carried out at the National Genomic Infrastructure SNP&SEQ Technology Platform, Uppsala, using an S1 flowcell, the NovaSeq 6000 system, and v1.5 sequencing chemistry (Illumina, Inc.).

### scRNA-seq analysis

Alignment of the scRNA-seq data to the reference genome mm10 was performed with CellRanger (version 3.1.0; Dobin et al., 2013; Zheng et al., 2017). Samples were then read into R (version 4.1.3; R Core Team, 2022) as Seurat objects (version 4.1.1; Hao et al., 2021). During processing of the data, cells with abnormally low or high UMI and gene counts indicative of debris and doublets were removed. Cells with mitochondrial gene content >5% were also removed. The thresholds were set by evaluating UMI and gene counts as well as mitochondrial gene content according to current best practices (Luecken and Theis, 2019). Cells identified as doublets using DoubletFinder (version 2.0.3; McGinnis et al., 2019) were also removed.

The data were normalized using the function NormalizeData (Seurat). Variable genes were detected using the FindVariableFeatures function (Seurat) with selection method set to “vst.” Gene expression was scaled while regressing out effects of cell cycle, UMI counts, and mitochondrial gene content. Cell cycle gene sets were obtained from the cell cycle genes list provided by Seurat and then converted to *Mus musculus* homologs. Principal component analysis and Uniform Manifold Approximation and Projection (UMAP) were carried out for dimensionality reduction, and clustering was performed with the Louvain algorithm (Seurat). After initial clustering, contaminating cells were removed and an additional round of clustering and dimensionality reduction was carried out on the remaining cells. DEGs were identified using the FindAllMarkers function (Seurat) with the test setting nonparametric Wilcoxon Rank Sum test. Clusters were combined in a semisupervised manner on the basis of the DEGs identified. Gene signatures were generated with the function AddModuleScore (Seurat) using the top five identified DEGs for each cluster. Pseudobulk differential gene expression analysis was carried out using DESeq2 (version 1.34.0) after generating a pseudobulk dataset for each sample (Love et al., 2014). The analysis was performed on each of the clusters generated through analysis of the scRNA-seq data. Shrinking of log_2_ fold changes was carried out using the LfcShrink function (DESeq2) with type set to “apeglm” (Zhu et al., 2019). Volcano plots of differential gene expression results were generated using EnhancedVolcano (version 1.12.0; Blighe et al., 2022). Significant DEGs were defined as adjusted P value <0.05 and a log_2_ fold change <−0.58 or >0.58. Heat maps of these genes were generated using the AverageExpression function (Seurat) on the scRNA-seq dataset using significant genes as input and taking the mean of the scale expressed for all the mice in the different groups.

### Data deposition

Accession number for scRNA-seq data: GSE218311.

### Statistical analysis

Statistical significance was determined by a Mann–Whitney U test using GraphPad Prism software (GraphPad).

### Online supplemental material

Fig. S1 shows representative flow cytometry plots of cDC subset composition in the MLN and SI-LP of *Xcr-cre*.DTA and ROSA-DTA mice and additional analysis of intestinal CD4^+^ and CD8^+^ T cell subset numbers in *Xcr1-cre.p28*^*fl/fl*^ and *p28*^*fl/fl*^ mice. Fig. S2 shows MLN cDC subset composition, intestinal histology, serum lipocalin-2 levels, and intestinal neutrophil numbers in 36-d-old *Xcr1-cre.p28*^*fl/fl*^ and *p28*^*fl/fl*^ mice. It shows *p28* expression by MLN cDC subsets in steady state and 5 and 12 h after in vivo administration of αCD40 and either Poly(I:C) or LPS. It shows numbers and proportions of cytokine-expressing OT-II cells in the MLN of recipient mice in the absence of immunization or following immunization with OVA, αCD40, and LPS. It shows the experimental outline for the in vivo αIL-27p28 neutralization studies and that αIL-27p28 reduces the proportion of IFNγ-expressing OT-II cells in the MLN of mice immunized with OVA, αCD40, and LPS. Fig. S3 shows Th subset locations within the SI-LP CD4^+^ T cell scRNA-seq UMAP as identified using lineage-associated gene expression analysis. It also shows heat maps of differential genes within each SI-LP CD4^+^ T cell cluster that differ between *Xcr1-cre.p28*^*fl/fl*^ and *p28*^*fl/fl*^ mice.

## References

[bib1] Anderson, D.A., III, C.-A. Dutertre, F. Ginhoux, and K.M. Murphy. 2021. Genetic models of human and mouse dendritic cell development and function. Nat. Rev. Immunol. 21:101–115. 10.1038/s41577-020-00413-x 32908299 PMC10955724

[bib2] Atarashi, K., T. Tanoue, M. Ando, N. Kamada, Y. Nagano, S. Narushima, W. Suda, A. Imaoka, H. Setoyama, T. Nagamori, . 2015. Th17 cell induction by adhesion of microbes to intestinal epithelial cells. Cell. 163:367–380. 10.1016/J.CELL.2015.08.058 26411289 PMC4765954

[bib3] Atarashi, K., T. Tanoue, T. Shima, A. Imaoka, T. Kuwahara, Y. Momose, G. Cheng, S. Yamasaki, T. Saito, Y. Ohba, . 2011. Induction of colonic regulatory T cells by indigenous *Clostridium* species. Science. 331:337–341. 10.1126/science.1198469 21205640 PMC3969237

[bib4] Blighe, K., S. Rana, and M. Lewis. 2022. EnhancedVolcano: Publication-ready volcano plots with enhanced colouring and labeling. R package version 1.14.0, https://github.com/kevinblighe/EnhancedVolcano.

[bib5] Cerovic, V., S.A. Houston, J. Westlund, L. Utriainen, E.S. Davison, C.L. Scott, C.C. Bain, T. Joeris, W.W. Agace, R.A. Kroczek, . 2015. Lymph-borne CD8α^+^ dendritic cells are uniquely able to cross-prime CD8^+^ T cells with antigen acquired from intestinal epithelial cells. Mucosal Immunol. 8:38–48. 10.1038/mi.2014.40 24850430 PMC4156465

[bib6] Demiri, M., K. Müller-Luda, W.W. Agace, and M. Svensson-Frej. 2017. Distinct DC subsets regulate adaptive Th1 and 2 responses during Trichuris muris infection. Parasite Immunol. 39:e12458. 10.1111/pim.12458 28802050

[bib7] Do, J.S., A. Visperas, Y.O. Sanogo, J.J. Bechtel, N. Dvorina, S. Kim, E. Jang, S.A. Stohlman, B. Shen, R.L. Fairchild, . 2016. An IL-27/Lag3 axis enhances Foxp3^+^ regulatory T cell-suppressive function and therapeutic efficacy. Mucosal Immunol. 9:137–145. 10.1038/mi.2015.45 26013006 PMC4662649

[bib8] Dobin, A., C.A. Davis, F. Schlesinger, J. Drenkow, C. Zaleski, S. Jha, P. Batut, M. Chaisson, and T.R. Gingeras. 2013. STAR: Ultrafast universal RNA-seq aligner. Bioinformatics. 29:15–21. 10.1093/bioinformatics/bts635 23104886 PMC3530905

[bib9] Goto, Y., C. Panea, G. Nakato, A. Cebula, C. Lee, M.G. Diez, T.M. Laufer, L. Ignatowicz, and I.I. Ivanov. 2014. Segmented filamentous bacteria antigens presented by intestinal dendritic cells drive mucosal Th17 cell differentiation. Immunity. 40:594–607. 10.1016/j.immuni.2014.03.005 24684957 PMC4084624

[bib10] Hall, A.O.H., D.P. Beiting, C. Tato, B. John, G. Oldenhove, C.G. Lombana, G.H. Pritchard, J.S. Silver, N. Bouladoux, J.S. Stumhofer, . 2012a. The cytokines interleukin 27 and interferon-γ promote distinct Treg cell populations required to limit infection-induced pathology. Immunity. 37:511–523. 10.1016/j.immuni.2012.06.014 22981537 PMC3477519

[bib11] Hall, A.O.H., J.S. Silver, and C.A. Hunter. 2012b. The immunobiology of IL-27. Adv. Immunol. 115:1–44. 10.1016/B978-0-12-394299-9.00001-1 22608254

[bib12] Hao, Y., S. Hao, E. Andersen-Nissen, W.M. Mauck III, S. Zheng, A. Butler, M.J. Lee, A.J. Wilk, C. Darby, M. Zager, . 2021. Integrated analysis of multimodal single-cell data. Cell. 184:3573–3587.e29. 10.1016/j.cell.2021.04.048 34062119 PMC8238499

[bib13] Hashimoto, K., S.K. Joshi, and P.A. Koni. 2002. A conditional null allele of the major histocompatibility IA-beta chain gene. Genesis. 32:152–153. 10.1002/gene.10056 11857806

[bib14] Hooper, K.M., J.-H. Yen, W. Kong, K.M. Rahbari, P.-C. Kuo, A.M. Gamero, and D. Ganea. 2017. Prostaglandin E2 inhibition of IL-27 production in murine dendritic cells: A novel mechanism that involves IRF1. J. Immunol. 198:1521–1530. 10.4049/jimmunol.1601073 28062696 PMC5296337

[bib15] Ivanov, I.I., K. Atarashi, N. Manel, E.L. Brodie, T. Shima, U. Karaoz, D. Wei, K.C. Goldfarb, C.A. Santee, S.V. Lynch, . 2009. Induction of intestinal Th17 cells by segmented filamentous bacteria. Cell. 139:485–498. 10.1016/j.cell.2009.09.033 19836068 PMC2796826

[bib16] Ivanov, I.I., R.L. Frutos, N. Manel, K. Yoshinaga, D.B. Rifkin, R.B. Sartor, B.B. Finlay, and D.R. Littman. 2008. Specific microbiota direct the differentiation of IL-17-producing T-helper cells in the mucosa of the small intestine. Cell Host Microbe. 4:337–349. 10.1016/j.chom.2008.09.009 18854238 PMC2597589

[bib17] Ivanov, I.I., T. Tuganbaev, A.N. Skelly, and K. Honda. 2022. T cell responses to the microbiota. Annu. Rev. Immunol. 40:559–587. 10.1146/annurev-immunol-10132010.1146/annurev-immunol-101320-011829 35113732 PMC9296687

[bib18] Joeris, T., C. Gomez-Casado, P. Holmkvist, S.J. Tavernier, A. Silva-Sanchez, L. Klotz, T.D. Randall, A.M. Mowat, K. Kotarsky, B. Malissen, and W.W. Agace. 2021. Intestinal cDC1 drive cross-tolerance to epithelial-derived antigen via induction of FoxP3^+^CD8^+^ T_regs_. Sci. Immunol. 6:eabd3774. 10.1126/sciimmunol.abd3774 34088744

[bib19] Joeris, T., K. Müller-Luda, W.W. Agace, and A.M.I. Mowat. 2017. Diversity and functions of intestinal mononuclear phagocytes. Mucosal Immunol. 10:845–864. 10.1038/mi.2017.22 28378807

[bib20] Kilgore, A.M., N.D. Pennock, and R.M. Kedl. 2020. cDC1 IL-27p28 production predicts vaccine-elicited CD8^+^ T cell memory and protective immunity. J. Immunol. 204:510–517. 10.4049/jimmunol.1901357 31871021 PMC6981069

[bib21] Kilgore, A.M., S. Welsh, E.E. Cheney, A. Chitrakar, T.J. Blain, B.J. Kedl, C.A. Hunter, N.D. Pennock, and R.M. Kedl. 2018. IL-27p28 production by XCR1^+^ dendritic cells and monocytes effectively predicts adjuvant-elicited CD8^+^ T cell responses. Immunohorizons. 2:1–11. 10.4049/immunohorizons.1700054 29354801 PMC5771264

[bib22] Kim, K.S., S.W. Hong, D. Han, J. Yi, J. Jung, B.G. Yang, J.Y. Lee, M. Lee, and C.D. Surh. 2016. Dietary antigens limit mucosal immunity by inducing regulatory T cells in the small intestine. Science. 351:858–863. 10.1126/science.aac5560 26822607

[bib23] Ladinsky, M.S., L.P. Araujo, X. Zhang, J. Veltri, M. Galan-Diez, S. Soualhi, C. Lee, K. Irie, E.Y. Pinker, S. Narushima, . 2019. Endocytosis of commensal antigens by intestinal epithelial cells regulates mucosal T cell homeostasis. Science. 363:eaat4042. 10.1126/science.aat4042 30846568 PMC6708280

[bib24] Lewis, K.L., M.L. Caton, M. Bogunovic, M. Greter, L.T. Grajkowska, D. Ng, A. Klinakis, I.F. Charo, S. Jung, J.L. Gommerman, . 2011. Notch2 receptor signaling controls functional differentiation of dendritic cells in the spleen and intestine. Immunity. 35:780–791. 10.1016/j.immuni.2011.08.013 22018469 PMC3225703

[bib25] Lin, C.H., M.C. Chen, L.L. Lin, D.A. Christian, B. Min, C.A. Hunter, and L.F. Lu. 2021. Gut epithelial IL-27 confers intestinal immunity through the induction of intraepithelial lymphocytes. J. Exp. Med. 218:e20210021. 10.1084/jem.20210021 34554189 PMC8480671

[bib26] Longhi, M.P., C. Trumpfheller, J. Idoyaga, M. Caskey, I. Matos, C. Kluger, A.M. Salazar, M. Colonna, and R.M. Steinman. 2009. Dendritic cells require a systemic type I interferon response to mature and induce CD4^+^ Th1 immunity with poly IC as adjuvant. J. Exp. Med. 206:1589–1602. 10.1084/jem.20090247 19564349 PMC2715098

[bib27] Love, M.I., W. Huber, and S. Anders. 2014. Moderated estimation of fold change and dispersion for RNA-seq data with DESeq2. Genome Biol. 15:550. 10.1186/s13059-014-0550-8 25516281 PMC4302049

[bib28] Lu, D.R., H. Wu, I. Driver, S. Ingersoll, S. Sohn, S. Wang, C.M. Li, and H. Phee. 2020. Dynamic changes in the regulatory T-cell heterogeneity and function by murine IL-2 mutein. Life Sci. Alliance. 3:e201900520. 10.26508/lsa.201900520 32269069 PMC7156283

[bib29] Luciani, C., F.T. Hager, V. Cerovic, and H. Lelouard. 2022. Dendritic cell functions in the inductive and effector sites of intestinal immunity. Mucosal Immunol. 15:40–50. 10.1038/s41385-021-00448-w 34465895

[bib30] Luda, K.M., T. Joeris, E.K. Persson, A. Rivollier, M. Demiri, K.M. Sitnik, L. Pool, J.B. Holm, F. Melo-Gonzalez, L. Richter, . 2016. IRF8 transcription-factor-dependent classical dendritic cells are essential for intestinal T cell homeostasis. Immunity. 44:860–874. 10.1016/j.immuni.2016.02.008 27067057

[bib31] Luecken, M.D., and F.J. Theis. 2019. Current best practices in single-cell RNA-seq analysis: A tutorial. Mol. Syst. Biol. 15:e8746. 10.15252/msb.20188746 31217225 PMC6582955

[bib32] Mattner, F., J. Magram, J. Ferrante, P. Launois, K. Di Padova, R. Behin, M.K. Gately, J.A. Louis, and G. Alber. 1996. Genetically resistant mice lacking interleukin-12 are susceptible to infection with Leishmania major and mount a polarized Th2 cell response. Eur. J. Immunol. 26:1553–1559. 10.1002/eji.1830260722 8766560

[bib33] Mayer, J.U., M. Demiri, W.W. Agace, A.S. MacDonald, M. Svensson-Frej, and S.W. Milling. 2017. Different populations of CD11b^+^ dendritic cells drive Th2 responses in the small intestine and colon. Nat. Commun. 8:15820. 10.1038/ncomms15820 28598427 PMC5472728

[bib34] McGinnis, C.S., L.M. Murrow, and Z.J. Gartner. 2019. DoubletFinder: Doublet detection in single-cell RNA sequencing data using artificial nearest neighbors. Cell Syst. 8:329–337.e4. 10.1016/j.cels.2019.03.003 30954475 PMC6853612

[bib35] Mei, Y., Z. Lv, L. Xiong, H. Zhang, N. Yin, and H. Qi. 2021. The dual role of IL-27 in CD4^+^T cells. Mol. Immunol. 138:172–180. 10.1016/j.molimm.2021.08.001 34438225

[bib36] Ohta, T., M. Sugiyama, H. Hemmi, C. Yamazaki, S. Okura, I. Sasaki, Y. Fukuda, T. Orimo, K.J. Ishii, K. Hoshino, . 2016. Crucial roles of XCR1-expressing dendritic cells and the XCR1-XCL1 chemokine axis in intestinal immune homeostasis. Sci. Rep. 6:23505. 10.1038/srep23505 27005831 PMC4804307

[bib37] Persson, E.K., H. Uronen-Hansson, M. Semmrich, A. Rivollier, K. Hägerbrand, J. Marsal, S. Gudjonsson, U. Håkansson, B. Reizis, K. Kotarsky, and W.W. Agace. 2013. IRF4 transcription-factor-dependent CD103(+)CD11b(+) dendritic cells drive mucosal T helper 17 cell differentiation. Immunity. 38:958–969. 10.1016/j.immuni.2013.03.009 23664832

[bib38] R Core Team. 2022. R: A language and environment for statistical computing. R Foundation for Statistical Computing, https://www.R-project.org/.

[bib39] Säwen, P., M. Eldeeb, E. Erlandsson, T.A. Kristiansen, C. Laterza, Z. Kokaia, G. Karlsson, J. Yuan, S. Soneji, P.K. Mandal, . 2018. Murine HSCs contribute actively to native hematopoiesis but with reduced differentiation capacity upon aging. Elife. 7:e41258. 10.7554/eLife.41258 30561324 PMC6298771

[bib40] Schlitzer, A., N. McGovern, P. Teo, T. Zelante, K. Atarashi, D. Low, A.W.S. Ho, P. See, A. Shin, P.S. Wasan, . 2013. IRF4 transcription factor-dependent CD11b^+^ dendritic cells in human and mouse control mucosal IL-17 cytokine responses. Immunity. 38:970–983. 10.1016/j.immuni.2013.04.011 23706669 PMC3666057

[bib41] Tanoue, T., K. Atarashi, and K. Honda. 2016. Development and maintenance of intestinal regulatory T cells. Nat. Rev. Immunol. 16:295–309. 10.1038/nri.2016.36 27087661

[bib42] Tindemans, I., M.E. Joosse, and J.N. Samsom. 2020. Dissecting the heterogeneity in T-cell mediated inflammation in IBD. Cells. 9:110. 10.3390/cells9010110 31906479 PMC7016883

[bib43] Trinchieri, G. 2003. Interleukin-12 and the regulation of innate resistance and adaptive immunity. Nat. Rev. Immunol. 3:133–146. 10.1038/nri1001 12563297

[bib44] Troy, A.E., C. Zaph, Y. Du, B.C. Taylor, K.J. Guild, C.A. Hunter, C.J.M. Saris, and D. Artis. 2009. IL-27 regulates homeostasis of the intestinal CD4^+^ effector T cell pool and limits intestinal inflammation in a murine model of colitis. J. Immunol. 183:2037–2044. 10.4049/jimmunol.0802918 19596985 PMC2821569

[bib45] Vignali, D.A.A., and V.K. Kuchroo. 2012. IL-12 family cytokines: Immunological playmakers. Nat. Immunol. 13:722–728. 10.1038/ni.2366 22814351 PMC4158817

[bib46] Voehringer, D., H.-E. Liang, and R.M. Locksley. 2008. Homeostasis and effector function of lymphopenia-induced “memory-like” T cells in constitutively T cell-depleted mice. J. Immunol. 180:4742–4753. 10.4049/jimmunol.180.7.4742 18354198 PMC2670614

[bib47] Welty, N.E., C. Staley, N. Ghilardi, M.J. Sadowsky, B.Z. Igyártó, and D.H. Kaplan. 2013. Intestinal lamina propria dendritic cells maintain T cell homeostasis but do not affect commensalism. J. Exp. Med. 210:2011–2024. 10.1084/jem.20130728 24019552 PMC3782055

[bib48] Wohn, C., V. le Guen, O. Voluzan, F. Fiore, S. Henri, and B. Malissen. 2020. Absence of MHC class II on cDC1 dendritic cells triggers fatal autoimmunity to a cross-presented self-antigen. Sci. Immunol. 5:eaba1896. 10.1126/sciimmunol.aba1896 32169954

[bib49] Yang, Y., M.B. Torchinsky, M. Gobert, H. Xiong, M. Xu, J.L. Linehan, F. Alonzo, C. Ng, A. Chen, X. Lin, . 2014. Focused specificity of intestinal TH17 cells towards commensal bacterial antigens. Nature. 510:152–156. 10.1038/nature13279 24739972 PMC4128479

[bib50] Yoshida, H., S. Hamano, G. Senaldi, T. Covey, R. Faggioni, S. Mu, M. Xia, A.C. Wakeham, H. Nishina, J. Potter, . 2001. WSX-1 is required for the initiation of Th1 responses and resistance to *L. major* infection. Immunity. 15:569–578. 10.1016/S1074-7613(01)00206-0 11672539

[bib51] Yoshida, H., and C.A. Hunter. 2015. The immunobiology of interleukin-27. Annu. Rev. Immunol. 33:417–443. 10.1146/annurev-immunol-032414-112134 25861977

[bib52] Yu, F., S. Sharma, J. Edwards, L. Feigenbaum, and J. Zhu. 2015. Dynamic expression of transcription factors T-bet and GATA-3 by regulatory T cells maintains immunotolerance. Nat. Immunol. 16:197–206. 10.1038/ni.3053 25501630 PMC4297509

[bib53] Zhang, H., A. Madi, N. Yosef, N. Chihara, A. Awasthi, C. Pot, C. Lambden, A. Srivastava, P.R. Burkett, J. Nyman, . 2020. An IL-27-driven transcriptional network identifies regulators of IL-10 expression across T helper cell subsets. Cell Rep. 33:108433. 10.1016/j.celrep.2020.108433 33238123 PMC7771052

[bib54] Zheng, G.X.Y., J.M. Terry, P. Belgrader, P. Ryvkin, Z.W. Bent, R. Wilson, S.B. Ziraldo, T.D. Wheeler, G.P. McDermott, J. Zhu, . 2017. Massively parallel digital transcriptional profiling of single cells. Nat. Commun. 8:14049. 10.1038/ncomms14049 28091601 PMC5241818

[bib55] Zhu, A., J.G. Ibrahim, and M.I. Love. 2019. Heavy-tailed prior distributions for sequence count data: Removing the noise and preserving large differences. Bioinformatics. 35:2084–2092. 10.1093/bioinformatics/bty895 30395178 PMC6581436

